# HIV risk perception and distribution of HIV risk among African, Caribbean and other Black people in a Canadian city: mixed methods results from the BLACCH study

**DOI:** 10.1186/1471-2458-13-184

**Published:** 2013-03-02

**Authors:** Shamara Baidoobonso, Greta R Bauer, Kathy Nixon Speechley, Erica Lawson

**Affiliations:** 1 Epidemiology & Biostatistics, The University of Western Ontario, London, ON, Canada; 2 Women’s Studies & Feminist Research, The University of Western Ontario, London, ON, Canada; 3 Regional HIV/AIDS Connection, London, ON, Canada; 4 London Cross Cultural Learner Centre, London, ON, Canada

**Keywords:** HIV/AIDS, African-descent, Black, HIV risk, Social determinants of health

## Abstract

**Background:**

African, Caribbean and other Black (ACB) people are a priority group for HIV prevention in Canada, but little is known about the epidemiology of HIV risk in this population. This paper helps fill the knowledge gap by: presenting service providers’ and ACB people’s perceptions about HIV risk in ACB populations; describing the distribution of HIV risk behaviours among ACB people according to markers of social status and position; and comparing results from these two analyses.

**Methods:**

The Black, African and Caribbean Canadian Health (BLACCH) Study is a mixed methods study that used semi-structured interviews and a cross-sectional quantitative questionnaire to collect information about HIV and health from 188 ACB people in London, Ontario, Canada. Qualitative content analysis was used to identify interview themes, and weighted bivariate statistical analyses were performed on the quantitative data. Behaviours related to HIV risk were stratified by sex, poverty status, immigration experience and employment status.

**Results:**

Community members perceived that they were at low risk for HIV and mainly focused on sexual risks. They called for more information about HIV in Canada and culturally appropriate HIV services. Service providers cited marital infidelity and cultural and religious attitudes about condoms as barriers to women protecting themselves. They mentioned cultural norms, beliefs about masculinity and underrepresentation of heterosexual ACB men at AIDS service organizations as barriers to men protecting themselves. There were few statistically significant differences in risk behaviours reported by men and women. Those living in poverty were more likely to abstain from sex (p = 0.006) and use condoms (p = 0.027) in the past year. Those living in Canada longer reported higher prevalences of forced sex (p < 0.001), mixing alcohol or drugs with sex (p = 0.001) and past STI diagnoses (p = 0.032). Stable employment was associated with higher prevalences of not using condoms in the past year (p = 0.005) and past STI diagnoses (p = 0.018).

**Conclusions:**

The results show that perceptions about ACB people’s HIV risk differ from actual risk, and those with higher social standing might be at greater risk. Furthermore, the social determinants of health are important factors in the epidemiology of HIV among ACB people.

## Background

People from countries where HIV is endemic are 12.6 times more likely to contract HIV through heterosexual exposure than other adults in Canada [1,2], and data show that Black people from Africa and the Caribbean account for 93% of people from endemic countries living with HIV [1,2]. In 2001, 55% of Canada’s Black population was foreign-born, and approximately 90% of Canada’s Black population had been in Canada for three generations or fewer [3]. Sub-Saharan African and Caribbean countries accounted for 73% of Black immigrants to Canada in 1961 and 94% in the period from 1991 to 2001 [3]. Due to the composition of Canada’s Black population, the term African, Caribbean and other Black (ACB) is used to recognize ethnic diversity and racial identity. In epidemiologic research on HIV in Canada, the terms “HIV-endemic” and “ACB” are used interchangeably because the two overlap substantially.

Although a priority group for HIV prevention in Canada, ACB people are under-researched and research on this population usually focuses on particular ethnic groups or Black men who have sex with men [4,5]. To the best of the authors’ knowledge, no quantitative studies have studied HIV risk in a broader Canadian ACB population. Furthermore, HIV surveillance data for ACB people are usually aggregated [1,6], thus masking variations in risk and influencing ACB people’s and service providers’ perceptions about how HIV risk is distributed among ACB people. This therefore impacts the uptake and delivery of prevention interventions [7], because perceptions influence attitudes, and attitudes influence behaviours [8]. Although these perceptions of risk may be valid, as they are sometimes based on observations that are akin to case studies, they may not accurately reflect the population’s risk profile, and their generalizability may be limited to particular groups of ACB people. It is therefore important to identify groups for which common perceptions about HIV risk may be valid, which can aid the design of more targeted HIV prevention interventions.

According to the World Health Organization, some social determinants of health (SDOH) are markers of social status and position (SSP) because they create power hierarchies, and SSP impacts individual behaviours, which are proximal risk factors for disease [9]. Markers of SSP include: race, sex/gender, poverty status/income, educational attainment, employment status, and immigration experience [9]. The first five are widely recognized as SDOH in the Canadian context [10], and immigration experience is gaining traction in Canada due to its relationship with social exclusion [11]. These SDOH have also been recognized as markers of SSP in the literature on Intersectionality Theory, which posits that markers of SSP act jointly to create unique social positions that influence behaviours and social and health-related outcomes [12,13].

Emerging literature shows that markers of SSP impact the distribution of HIV risk, but most research in this area has been conducted in Sub-Saharan Africa. There, research findings show that poverty status/income [14-16], educational attainment [17,18], and employment status [19,20] impact HIV risk. Studies from North America and Western Europe show that in ACB populations, the epidemiology of HIV is impacted by sex/gender [21-24], and immigration experience [25-28]. Results vary among studies, so the magnitudes and directions of these relationships are unclear. However, this is not surprising, as the impacts of markers of SSP are context-specific. To date, no study from North America has looked at multiple markers of SSP within a broad ACB population, so this exploratory study is the first of its kind.

This paper has three objectives. First, this paper will qualitatively present perceptions some ACB people and service providers have about HIV risk and protective behaviours within ACB populations. Second, this paper will use markers of SSP to quantitatively describe the distribution of HIV risk and protective behaviours and identify groups of ACB people who might be at increased risk for HIV exposure and transmission. Third, this paper will compare perceptions presented under the first objective to the risk profile presented under the second objective.

## Methods

### Setting

The data in this paper were gathered as part of the Black, African and Caribbean Canadian Health (BLACCH) Study, conducted in London, Canada. London is in southwestern Ontario, located midway between Toronto, Canada and Detroit, United States of America. It is an urban area surrounded by farmlands, with a population of about 370,000 people [29]. Black Londoners comprise approximately 2% of the population (~7,500 people), and about 61% of this population (~4,500 people) is aged 18 or older.

### Research approach

This study used a community-based research (CBR) approach, which is recognized and promoted for conducting HIV research with practical implications. CBR seeks to combine rigorous research methods with equitable partnerships between academic researchers and communities affected by the research [30]. It incorporates multiple sources of knowledge that can influence policy and the delivery of health programs and services [30,31]. Furthermore, CBR aims to strengthen communities affected by the research by raising questions that are of interest to them [32]. At its best, CBR ensures that research is scientifically sound, relevant and ethical [30-32]. Partner organizations for the BLACCH Study were: The University of Western Ontario, the Regional HIV/AIDS Connection, and the London Cross Cultural Learner Centre—a research university, an AIDS service organization, and a settlement organization, respectively. The research team also included community members not affiliated with these three organizations. Community members on the research team included African and Caribbean men and women whose ages ranged from the mid-twenties to late-forties, and they were students, entrepreneurs and people employed by the aforementioned organizations. With the exception of one person, all were immigrants or refugees, and the younger community members on the team had been raised in Canada or the United States of America.

### Research design

The BLACCH Study had two phases. In Phase I, research team members conducted qualitative semi-structured interviews, which contributed to the development of the quantitative questionnaire used in Phase II. Data from both phases were triangulated to meet this paper’s objectives.

### Interview sample and procedures

Three ACB women on the research team conducted one-on-one semi-structured interviews with a purposive sample of eight service providers and 22 ACB people aged 16 or older. The interview participants were recruited in London, Ontario from September 2009 to February 2010. Purposive sampling was used because it allows researchers to reach a diverse sample and gather information about a breadth of experiences [33]. The service providers included in the sample were front-line workers from AIDS service organizations, clinics, settlement organizations and community organizations that had ACB clients. Whenever possible, ACB service providers were selected and interviewed based on their experiences as service providers. The community members were chosen based on: age, HIV status, injection drug use history, sexual orientation, income, immigration experience, religion, ethnicity and sex.

Participants completed a demographics questionnaire, followed by an interview about health and HIV. Community members were asked questions about personal and community risks, HIV service needs and myths about HIV. Service providers were asked about barriers to HIV prevention for ACB men and women. The interviews were audio-recorded and lasted from 30 minutes to over 2 hours, with most lasting approximately 40 minutes. Upon completing the interviews, community member participants were offered $10 and a list of local organizations where they could access services.

### Qualitative analysis

Two research team members checked the interview transcripts for errors and corrected them as needed. SB and another interviewer analyzed the transcripts using qualitative content analysis (QCA), which identifies a broad range of themes, thereby making it complementary to purposive sampling [34,35], which also has the goal of capturing a breadth of information. QCA is also an appropriate method to use when little data are available in a particular area, and it consists of identifying themes that emerge from the data [36]. SB and another interviewer independently completed initial coding of the interviews, and the two met for debriefing sessions to compare their notes and summaries and record insights they gained from the data. They identified emergent themes on a question-by-question basis. SB looked for patterns and areas in which generalizations could be made and re-examined these generalizations based on new and existing knowledge from community members and the literature.

### Questionnaire sample and procedures

Following the interviews, the research team recruited ACB people to participate in Phase II of the study from November 2010 to November 2011. The inclusion criteria for the self-administered questionnaire survey were as follows: 18 years or older, self-identify as Black, and live or spend most of the year in London or Middlesex County. Interview participants were recruited using a combination of venue-based sampling, snowballing, outreach and a media campaign in order to overcome some of the weaknesses associated with each sampling method and reach a broad, diverse sample. These methods have been used successfully to recruit similar populations into health research [37-39]. Outreach and venue-based sampling took place at schools, community organizations, summer festivals, libraries, sporting events, and public spaces. A very small, diverse group of participants recruited others into the study. The media campaign included posters, interviews on local radio shows, and radio advertisements. While only one participant was directly recruited through the media campaign, it appeared to have increased familiarity with the study, since some people who were approached at the different venues said that they were already aware of the study.

Recruitment procedures were developed based on Dillman’s “Tailored Design Method” for mailed surveys [40]. To build trust and provide non-monetary rewards, the research team recruited participants face-to-face through one-on-one contact, and if interested, participants could request copies of the final project report. Social costs to participation were reduced through using simple language in the questionnaire, providing it in a portable format, and providing an addressed, stamped return envelope. Potential participants provided their contact information, and SB called or e-mailed biweekly with reminders.

The questionnaire consisted of seven sections that covered topics that included health behaviours, sexual health and HIV service needs. Individuals who pre-tested the survey completed it in 25 minutes to one hour, and most said it took them approximately 30 minutes to complete the survey in one sitting. It is likely that participants spent as much time completing the survey. Participants were told that consent was implied when the questionnaire was returned.

In all, 595 questionnaires were distributed, and 188 (32%) were returned. Although low, this response rate is not unusual for a study focusing on an ACB population in Ontario, Canada [5]. The response rate was impacted by some aspects of the research project. Based on conversations with three community members during follow-up phone calls and e-mails, some people did not participate in the research because they were uncomfortable answering questions about sexual behaviours. Several others said they did not have time to complete the survey, so the length of the questionnaire might have also played a role. The survey was offered in English and French, but many community members have a first language other than English or French, and they might be more comfortable completing a survey in their native language. Lastly, based on a comparison between the sample and the underlying population (as defined by the 2006 Census), survey participants were more educated than the local Black population (*χ*^2^ = 164.06, df = 8, p < 0.0001). Survey participants were required to read a large volume of information in the survey packages (i.e. information and consent letter, list of service organizations, introductory and instruction letter), so literacy may have also impacted the response rate.

### Measures

The self-administered questionnaire covered a variety of health topics. However, only questions related to markers of SSP and HIV exposure and transmission were included in this analysis. Sex, poverty status, immigration experience, and employment status were the markers of SSP on which these analyses focus. Poverty status was defined using the low-income cut-off (LICO) score, which is based on household income and the number of people supported by it [41]. Immigration experience was divided into length of time in Canada and immigration status at time of survey completion. Risk and protective behaviours assessed included HIV testing, abstinence, number of sex partners, condom use with various types of partners, being in a non-monogamous sexual partnership, sharing injection drug use equipment, age of sexual debut, ever mixing intercourse with alcohol or drugs, having a partner who had ever used injection drugs, having a history of other sexually transmitted infections (STIs), being pressured to engage in intercourse, and ever engaging in transactional sex.

### Statistical analysis

Data from the questionnaires were analyzed using SAS software, version 9.3 [42]. To adjust for selection bias due to convenience sampling, the sample was compared to the Black population in London on age, educational attainment, sex and ethnicity using the 2006 Census. Chi-square tests showed that the two groups were significantly different at the p = 0.01 level on all four characteristics. Hence, non-response weights were derived using these four variables in a logistic regression model in which being included in the sample was the outcome [43,44]. Predicted probabilities from this model were used to calculate the weights, which were normalized so that they summed to 188. The mean and standard deviation of the weights were 1.00 and 1.13, respectively. Continuous variables were categorized for the descriptive analyses, risk factors were stratified according to the markers of SSP, and point prevalences and 95% Wilson confidence intervals were calculated along with Rao-Scott chi-square tests to assess the relationships between markers of SSP and risk factors used in these analyses. The analyses were performed using the weights in the SURVEYFREQ procedure in the SAS software.

### Data integration

The mixed methods, community-based approach to this research helps to produce a more complete picture of the epidemiology of HIV in London’s ACB population. Qualitative and quantitative data were combined using concurrent triangulation [45]. Both sets of data were collected and analyzed separately and integrated by comparing them and noting areas where they converged and failed to converge. These results are part of the discussion section of this paper.

### Ethical approval and consent

This research was approved by the Non-Medical Research Ethics Board at The University of Western Ontario. Standard consent procedures were followed.

## Results

### Phase I

#### Description of the interview sample

As expected, the sample was diverse. The median age for community members was 41 years, with the youngest being 16 years old. For service providers, the median age was 49 years. People with African and Caribbean ethnicity accounted for 45% and 36% of the community members, respectively. Among service providers, they accounted for 50% and 13% of the sample, respectively. Women represented 55% of the sample among community members, and 75% of the service providers. It was difficult to find male service providers, because there were few men working in the organizations contacted, and even fewer male service providers worked with ACB clients extensively enough to participate in this research. Among community members and service providers, people of Christian faith were the majority, and Muslims were represented in both groups. The majority of community members were foreign-born, and regardless of birthplace, the majority were Canadian citizens. Community members showed a range of educational levels and household incomes, and all the service providers had at least some post-secondary education. While community members reported a range of relationship statuses and sexual orientations, all service providers reported that they were heterosexuals in stable relationships.

#### Perception of low personal risk

Regardless of ethnicity, when community members thought about HIV risk, they thought of it as something that was removed from Canada and happens elsewhere. When acknowledging the presence of HIV in Canada, HIV was seen as something that affects others, but not them personally.

Most community members said HIV was a problem in their communities. One African community member talked about having relatives in Africa who died as a result of HIV and many people in the community in Canada being unaware of their HIV status. However, some Caribbean community members and one African either said HIV was not a problem in Canada, or that they did not know if HIV was a problem in their communities. Although the interviews were specifically about the Canadian context, some individuals talked about HIV in African countries.

*…[I]t affected me a lot back home like… our friends…they go back home and… it’s easy to get contracted with HIV….* [African female]

Almost unanimously, community members said their personal risk for contracting HIV was low or non-existent but acknowledged that it was difficult to be certain about one’s risk. Some women said they were not at risk due to abstinence or marriage. On the other hand, while men said they had low risk of HIV infection, none said he had no risk, and none cited abstinence or marriage as reasons for having low HIV risk. At the same time, community members also talked about high risk for HIV in their communities, in general.

*I don’t believe I’m at risk for that. My greatest risk would be whether or not my husband had sexual intercourse with people I don’t know about…* [Canadian/Caribbean female]

*[Y]ou never know, but I think it’s zero because… I am like very careful…* [African male]

#### Risk behaviours

Community members generally focused on sexual risk behaviours related to HIV infection and largely ignored non-sexual modes of exposure or transmission. They cited relationship factors, such as being in a non-monogamous sexual relationship, not knowing a partner’s sexual history, and general lack of education about safer sex and HIV prevention as risks for HIV infection. There was a gender split in these responses—while male and female participants talked about the relationship aspects of risk, only women mentioned alcohol and injection drug use.

*…[F]or those that drink…they can’t say no, they just go on and do whatever comes to their mind so mostly it’s through sexual activities….Of course there is also a substance, injection drugs.* [African female]

*I would say a lot of factors; the first one would be unprotected sex, another one would be not knowing the sexual background of your partner.* [Caribbean male]

#### Services to meet HIV-related needs

When asked about the types of services they believed ACB people require to meet their HIV-related needs, the majority of community members called for more information and education about HIV in Canada. Additionally, one participant called for more condoms in the general community, not just at HIV testing sites. HIV testing, especially testing as couples and families, was cited by several community members. Many also believed structural factors need to be addressed in order to better meet ACB people’s HIV prevention needs. For instance, they said that culturally appropriate services designed to specifically target ACB people and address the unique realities of their lives need to be provided, community-based programs need to be developed and supported, and access to care and greater sensitization around HIV are needed.

*… [S]omething set up where the youth can go and have these classes that teach them about HIV/AIDS and the prevalence, the current status of like HIV prevalence in their community so that they can be aware of it…more testing centers that are not out in the public… obviously provide condoms for people who can’t really practice abstinence…* [African female]

*[I]t is obvious that there are some services here in the city and I don’t think the service is for everybody in my view…. Again when I see the health care delivery system I don’t find it is geared…like say in this community, on a culturally sensitive area except for Native Canadians.* [Caribbean male]

#### Barriers that prevent women from protecting themselves

Service providers offered a variety of potential reasons for women not protecting themselves. Some said the need for love or acceptance in the context of sexual and marital relationships was a barrier to ACB women protecting themselves from HIV infection. Also, lack of empowerment among women was seen to manifest into lack of ability to negotiate condom use, intimate partner violence, and abuse in general. Other barriers cited included: marital infidelity, ACB women’s trust in their sexual partners, and cultural and religious attitudes discouraging condom use and communication about sex and safer sex practices. Lastly, service providers said women’s ignorance about HIV in their communities and lack of education about how to protect themselves were potential barriers.

*…[T]he need to be accepted, the need to be loved, the need to feel someone wants to be with me, someone thinks I’m attractive and somehow better judgment saying, “I need to take protection”, doesn’t happen…* [Female service provider]

*…[M]aybe ignorance if they don’t know…that is really a problem. Otherwise I think if any woman would know there is… HIV she would protect herself.* [Male service provider]

#### Barriers that prevent men from protecting themselves

For ACB men, service providers cited lack of condom use as a barrier to protection from HIV infection. Additionally, they said ACB men faced barriers related to the expression of masculinity, such as being less likely to access services than ACB women and believing that they cannot control themselves sexually. The service providers mentioned cultural norms and beliefs dictating that ACB men not disclose information, and they perceived that ACB men generally did not seek information because they were expected to be knowledgeable about everything. They also mentioned barriers for specific groups of men—some male injection drug users sharing drug use equipment, some gay men having a sense of “fatalism”, and the hierarchy of beauty in gay culture preventing some gay men from protecting themselves. Service providers reported that some heterosexual men believe that they cannot become infected. However, these men are generally not reached by HIV prevention messages, and they are unlikely to access HIV/AIDS services due to the underrepresentation of heterosexual ACB male staff in AIDS service organizations.

*I think … that notion hasn’t been engrained in them that condoms are important and… I’m not even talking about the transmission through intravenous drug use and sharing of… drug paraphernalia use.* [Female service provider]

### Phase II

#### Description of the questionnaire sample

The characteristics of the 188 participants recruited for Phase II are provided elsewhere [46]. Their ages ranged from 18 to over 72 years, and 11% did not identify as heterosexual. Half had never been married, and 32% were married or living common-law. The majority (80%) identified as Christian, and 5% identified as Muslim. The sample included a variety of ethnic identities—57% identified with an African ethnicity, 38% identified as Caribbean, 3% were multi-generational Canadians, and 2% had other ethnic identities. Women outnumbered men (60% versus 40%), 70% of participants were above the LICO, over 80% had higher than a high school education, and 42% reported being in school at the time they completed the questionnaire. The study was conducted during an economic recession, so some people were in school preparing for a “second career,” some were regulated professionals studying for Canadian licenses, some were learning English, and some were completing their educations. Additionally, 15% (29/188) of participants reported that they were born in Canada.

#### Sex

Table 1 displays results comparing males and females. Women were more likely to have experienced a history of forced or unwanted sex (χ_RS_^2^ = 3.39, df = 4, p = 0.033). On the other hand, women were less likely to ever mix sex with drugs or alcohol (χ_RS_^2^ = 3.89, df = 1, p = 0.049) or have two or more sex partners in the last 12 months (χ_RS_^2^ = 9.96, df = 3, p = 0.019).

**Table 1:** Weighted prevalences for risk factors for HIV infection by sex

**Risk factors**	**Female (n = 113)**	**Male (n = 75)**	**P-value**
	**wPrev (95% CI)**	**wPrev (95% CI)**	
*Factors associated with exposure to HIV*			
Age of sexual debut			0.494^a^
Never had sex	14.1 (8.9, 21.8)	11.0 (4.3, 25.7)	
12 years old or younger	5.3 (1.4, 17.6)	8.9 (3.1, 23.3)	
13 to 15 years old	9.2 (4.7, 17.5)	10.3 (4.4, 22.0)	
16 to 18 years old	34.9 (24.0, 47.6)	22.9 (13.5, 36.2)	
19+ years old	24.4 (16.8, 34.1)	38.7 (23.2. 57.0)	
Engaged in transactional sex	5.5 (2.2, 13.1)	——	0.107^b^
History of forced/unwanted sex	31.8 (21.7, 44.0)	10.1 (3.9, 23.7)	**0.033**^a^*
Had a sexual partner who injected drugs	3.7 (1.2, 11.2)	0.8 (0.1, 6.3)	0.167^a^
*Factors associated HIV exposure and transmission*			
Ever test for HIV	56.5 (44.4, 67.9)	63.0 (47.1, 76.6)	0.706^a^
HIV test in Canada, past year	15.0 (9.4, 23.3)	22.4 (11.3, 39.7)	0.442^a^
Shared drug use equipment	1.5 (0.3, 7.9)	——	0.380^b^
Abstinence, lifetime	14.1 (8.9, 21.8)	11.0 (4.3, 25.7)	0.647^a^
Abstinence, past year	32.2 (22.5, 43.6)	20.0 (10.5, 34.8)	0.179^a^
Unprotected sex, cohabiting regular partner, past year	50.9 (39.0, 62.6)	48.5 (32.6, 64.7)	0.644^a^
Unprotected sex, non-cohabiting regular partner, past year	41.2 (30.2, 53.2)	38.5 (23.7, 55.9)	0.544^a^
Unprotected sex during last intercourse, regular partner	39.2 (28.4, 51.2)	40.2 (25.2, 57.3)	0.928^a^
Unprotected sex, casual partner, past year	11.5 (4.8, 25.2)	6.7 (2.0, 20.6)	0.608^a^
Unprotected sex during last intercourse, casual partner	6.8 (2.1, 19.7)	17.6 (6.3, 40.2)	0.152^a^
Never using condom, past year	38.4 (27.1, 51.1)	35.0 (22.3, 50.2)	0.730^a^
Ever mixed sex with drugs or alcohol	26.9 (18.5, 37.4)	43.8 (28.5, 60.3)	**0.049**^a^*
Non-monogamous sexual partnership, past year	10.6 (5.4, 19.7)	24.1 (12.2, 42.0)	0.068^a^
History of sexually transmitted infections	27.0 (17.5, 39.0)	17.2 (8.6, 31.2)	0.735^a^
Number of sex partners, lifetime			0.531^a^
None	14.1 (8.9, 21.8)	11.0 (4.3, 25.7)	
1	5.8 (2.8, 11.8)	12.3 (3.3, 36.4)	
2 to 4	23.6 (15.9, 33.5)	13.9 (7.6, 24.3)	
5 to 9	18.3 (9.7, 32.0)	18.6 (9.9, 32.3)	
10 to 19	9.2 (4.6, 17.7)	20.2 (9.4, 38.3)	
20 or more	9.7 (4.2, 20.7)	11.5 (4.8, 25.0)	
Number of sex partners, past year			**0.019**^a^*
0	32.2 (22.5, 43.6)	20.0 (10.5, 34.8)	
1	44.7 (33.2, 56.7)	30.5 (19.0, 45.0)	
2	12.0 (6.0, 22.6)	30.2 (15.7, 50.2)	
3 or more	5.6 (2.6, 11.7)	16.0 (7.2, 31.7)	

n = column total, not adjusted for nonresponse using sample weights.

^a^ P-value from Rao-Scott chi-square test.

^b^ P-value from Rao-Scott chi-square test with assumed design correction of 2 (conservative estimate).

* Statistically significant at p = 0.05.

#### Poverty status

People living at or below the LICO appear to have a lower HIV risk profile compared to those living above the LICO (Table 2). People living at or below the LICO were significantly less likely to have a history of forced or unwanted sex (χ_RS_^2^ = 6.34, df = 1, p = 0.011) or not use condoms in the past year (χ_RS_^2^ = 4.88, df = 1, p = 0.027). When partner types were considered, there was no significant difference in having unprotected sex with casual partners when comparing people living at or below the LICO to people living above it (χ_RS_^2^ = 2.51, df = 1, p = 0.113). However, people living at or below the LICO were significantly less likely to have unprotected sex with cohabiting (χ_RS_^2^ = 11.97, df = 1, p = 0.001) and non-cohabiting (χ_RS_^2^ = 12.96, df = 1, p < 0.001) regular partners. They were also significantly less likely to have had unprotected sex during their last intercourse with a regular partner (χ_RS_^2^ = 5.76, df = 1, p = 0.016). In addition to their condom use, people living below the LICO appeared to be at lower risk for HIV exposure and transmission because they were more likely to have never had sex (χ_RS_^2^ = 6.00, df = 1, p = 0.014) and abstain from sex in the past year (χ_RS_^2^ = 7.55, df = 1, p = 0.006).

**Table 2:** Weighted prevalences for risk factors for HIV infection by poverty status

**Risk factors**	**At or below LICO (n = 53)**	**Above LICO (n = 122)**	**P-value**
	**wPrev (95% CI)**	**wPrev (95% CI)**	
*Factors associated with exposure to HIV*			
Age of sexual debut			0.357^a^
Never had sex	23.9 (11.0, 44.3)	7.5 (4.0, 13.5)	
12 years old or younger	5.6 (1.23, 21.8)	8.9 (3.3, 21.7)	
13 to 15 years old	8.7 (3.6, 19.4)	11.6 (5.8, 21.7)	
16 to 18 years old	30.9 (16.9, 49.6)	25.4 (16.6, 36.7)	
19+ years old	29.3 (13.8, 51.9)	34.0 (21.6, 49.0)	
Engaged in transactional sex	4.1 (1.2, 13.2)	2.2 (0.6, 8.2)	0.583^a^
History of forced/unwanted sex	9.8 (4.3, 20.8)	25.0 (15.6, 37.5)	**0.011**^a^*
Had a sexual partner who injected drugs	1.4 (0.2, 9.2)	2.7 (0.8, 8.5)	0.565^a^
*Factors associated HIV exposure and transmission*			
Ever test for HIV	55.0 (35.7, 72.9)	61.2 (48.3, 72.6)	0.702^a^
HIV test in Canada, past year	22.7 (8.8, 47.1)	16.2 (9.3, 26.8)	0.510^a^
Shared drug use equipment	——	1.1 (0.2, 6.0)	0.581^b^
Abstinence, lifetime	23.9 (11.0, 44.3)	7.5 (4.0, 13.5)	**0.014**^a^*
Abstinence, past year	41.2 (24.1, 60.7)	16.3 (10.5, 24.5)	**0.006**^a^*
Unprotected sex, cohabiting regular partner, past year	27.9 (15.7, 44.6)	59.9 (46.6, 71.9)	**0.001**^a^*
Unprotected sex, non-cohabiting regular partner, past year	18.1 (9.0, 33.0)	48.8 (35.7, 62.0)	**<0.001**^a^*
Unprotected sex during last intercourse, regular partner	24.0 (13.0, 40.1)	46.0 (33.0, 59.6)	**0.016**^a^*
Unprotected sex, casual partner, past year	4.8 (1.5, 14.2)	11.4 (4.7, 25.1)	0.113^a^
Unprotected sex during last intercourse, casual partner	6.6 (2.0, 19.3)	16.9 (6.6, 37.0)	0.217^a^
Never using condom, past year	21.9 (11.3, 38.2)	44.0 (32.4, 56.4)	**0.027**^a^*
Ever mixed sex with drugs or alcohol	34.3 (17.8, 55.7)	38.8 (27.0, 52.0)	0.664^a^
Non-monogamous sexual partnership, past year	29.8 (13.8, 53.0)	12.5 (5.9, 24.5)	0.114^a^
History of sexually transmitted infections	14.3 (6.9, 27.3)	27.4 (17.5, 40.2)	0.109^a^
Number of sex partners, lifetime			0.312^a^
None	23.9 (11.0, 44.4)	7.5 (4.0, 13.5)	
1	4.7 (1.5, 14.1)	12.6 (4.1, 32.6)	
2 to 4	21.6 (11.7, 36.5)	16.0 (10.2, 24.2)	
5 to 9	13.9 (5.2, 32.2)	18.7 (10.8, 30.3)	
10 to 19	19.2 (6.2, 45.9)	14.7 (7.7, 26.5)	
20 or more	10.3 (3.8, 25.1)	12.2 (5.7, 24.1)	
Number of sex partners, past year			0.146^a^
0	41.2 (24.1, 60.7)	16.3 (10.5, 24.5)	
1	25.2 (13.8, 41.5)	42.8 (30.8, 55.8)	
2	20.7 (7.1, 47.0)	23.5 (12.3, 40.2)	
3 or more	12.9 (5.7, 26.7)	11.8 (5.0, 25.4)	

n = column total, not adjusted for nonresponse using sample weights.

^a^ P-value from Rao-Scott chi-square test.

^b^ P-value from Rao-Scott chi-square test with assumed design correction of 2 (conservative estimate).

*Statistically significant at p = 0.05.

#### Time in Canada

HIV risk may be related to the amount of time a person has lived in Canada (Table 3). Overall, immigrants appeared to be at lower risk for HIV exposure and transmission than Canadian-born persons. For instance, immigrants were significantly less likely to report: having a history of forced or wanted sex (χ_RS_^2^ = 24.73, df = 3, p < 0.001), ever mixing sex with drugs or alcohol (χ_RS_^2^ = 15.99, df = 3, p = 0.001), having a history of STIs (χ_RS_^2^ = 8.78, df = 3, p = 0.032), having a higher number of sex partners in their lifetimes (χ_RS_^2^ = 28.08, df = 15, p = 0.021) or having a higher number of sex partners in the past year (χ_RS_^2^ = 25.44, df = 9, p = 0.003). However, as the length of time in Canada increased, immigrants’ risk profile more closely mimicked that of born Canadians. As the time spent in Canada increased, immigrants were more likely to report: having a history of forced or unwanted sex, having unprotected sex with a regular or casual partner in the past 12 months, not using a condom in the past 12 months, ever mixing sex with drugs or alcohol, having a history of STIs, ever having sex or having sex in the past year.

**Table 3:** Weighted prevalences for risk factors for HIV infection by time in Canada

**Risk factors**	**0-5 year (n = 45)**	**>5 to 15 years (n = 51)**	**>15 years (n = 57)**	**Canadian-born (n = 29)**	**P-value**
	**wPrev (95% CI)**	**wPrev (95% CI)**	**wPrev (95% CI)**	**wPrev (95% CI)**	
*Factors associated with exposure to HIV*					
Age of sexual debut					0.070^a^
Never had sex	26.7 (14.5, 43.8)	21.8 (8.4, 45.7)	3.7 (1.1, 12.2)	6.9 (1.9, 21.9)	
12 years old or younger	9.3 (2.2, 32.2)	1.3 (0.2, 9.3)	13.4 (4.3, 34.5)	5.3 (1.0, 23.4)	
13 to 15 years old	8.6 (3.0, 22.4)	8.3 (3.1, 20.2)	10.3 (3.3, 27.7)	17.1 (7.1, 36.0)	
16 to 18 years old	32.7 (17.4, 52.7)	17.8 (8.5, 33.5)	37.7 (23.6, 54.3)	31.3 (14.8, 54.4)	
19+ years old	19.3 (10.4, 33.1)	44.4 (22.8, 68.2)	22.4 (12.8, 36.3)	37.6 (18.2, 62.0)	
Engaged in transactional sex	——	3.0 (0.7, 11.8)	1.3 (0.2, 8.5)	8.5 (2.3, 26.6)	0.425^b^
History of forced/unwanted sex	11.5 (4.3, 27.4)	9.4 (3.7, 21.7)	16.0 (8.5, 27.9)	58.5 (37.2, 77.0)	**<0.001**^a^*
Had a sexual partner who injected drugs	——	——	1.0 (0.1, 8.0)	11.0 (3.7, 28.8)	0.113^b^
*Factors associated HIV exposure and transmission*					
Ever test for HIV	76.1 (60.4, 86.9)	60.1 (37.8, 78.8)	41.5 (26.6, 58.2)	72.0 (51.5, 86.1)	0.131^a^
HIV test in Canada, past year	21.0 (11.5, 35.0)	15.0 (7.4, 28.0)	17.7 (7.6, 36.0)	7.4 (2.1, 22.6)	0.551^a^
Shared drug use equipment	——	——	——	4.2 (0.8, 19.5)	0.392^b^
Abstinence, lifetime	26.7 (14.5, 43.8)	21.8 (8.4, 45.7)	3.7 (1.1, 12.2)	6.9 (1.9, 21.9)	**0.001**^a^*
Abstinence, past year	52.0 (34.6, 69.0)	32.9 (16.2, 55.4)	20.4 (10.5, 36.0)	10.0 (3.4, 25.9)	**0.011**^a^*
Unprotected sex, cohabiting regular partner, past year	28.7 (16.0, 46.1)	51.6 (29.7, 72.9)	67.0 (49.6, 80.7)	44.9 (25.4, 66.2)	0.090^a^
Unprotected sex, non-cohabiting regular partner, past year	17.5 (8.0, 34.0)	43.4 (22.0, 67.5)	54.1 (37.7, 69.7)	38.1 (20.6, 59.3)	0.101^a^
Unprotected sex during last intercourse, regular partner	20.1 (10.2, 35.7)	50.3 (28.5, 72.1)	55.1 (38.6, 70.6)	25.8 (12.5, 45.9)	**0.027**^a^*
Unprotected sex, casual partner, past year	3.5 (0.8, 13.4)	2.2 (0.4, 10.7)	15.1 (5.5, 35.2)	5.5 (1.3, 20.0)	0.056 ^a^
Unprotected sex during last intercourse, casual partner	——	25.2 (7.3, 59.2)	10.3 (2.9, 30.3)	5.5 (1.3, 20.0)	0.066 ^b^
Never using condom, past year	20.6 (10.5, 36.6)	31.1 (17.9, 48.3)	50.8 (34.0, 67.5)	43.6 (23.5, 66.0)	0.103^a^
Ever mixed sex with drugs or alcohol	14.3 (6.7, 28.0)	19.8 (9.8, 36.0)	43.6 (28.2, 60.4)	63.9 (40.4, 82.1)	**0.001**^a^*
Non-monogamous sexual partnership, past year	15.7 (5.7, 36.7)	7.3 (2.2, 21.2)	18.1 (7.7, 37.2)	16.5 (6.5, 36.0)	0.644^a^
History of sexually transmitted infections	6.1 (2.0, 17.1)	13.3 (5.7, 28.2)	31.4 (17.7, 49.3)	42.3 (23.1, 64.1)	**0.032**^a^*
Number of sex partners, lifetime					**0.021**^b^*
None	26.7 (14.5, 43.8)	21.8 (8.4, 45.7)	3.7 (1.1, 12.2)	6.9 (1.9, 21.9)	
1	1.1 (0.1, 9.8)	24.2 (6.7, 58.6)	9.2 (4.0, 19.4)	——	
2 to 4	24.3 (13.5, 39.7)	21.9 (11.2, 38.5)	20.7 (11.3, 35.0)	6.6 (1.8, 21,5)	
5 to 9	22.7 (10.0, 43.8)	8.5 (3.3, 20.4)	26.0 (13.4, 44.2)	23.4 (9.2, 48.1)	
10 to 19	3.3 (0.8, 13.2)	8.1 (2.5, 23.3)	8.6 (3.2, 21.2)	34.3 (15.9, 59.1)	
20 or more	7.9 (1.5, 32.4)	3.5 (0.9, 12.6)	18.3 (7.8, 37.3)	14.4 (5.5, 32.6)	
Number of sex partners, past year					**0.003 **^a^*
0	52.0 (34.6, 69.0)	32.9 (16.2, 55.4)	20.4 (10.5, 36.0)	10.0 (3.4, 25.9)	
1	27.4 (15.3, 44.2)	24.8 (13.0, 42.2)	47.1 (31.4, 63.3)	56.9 (34.6, 76.8)	
2	10.1 (2.6, 32.2)	35.0 (14.7, 62.8)	18.3 (8.5, 34.9)	3.9 (0.7, 17.8)	
3 or more	6.8 (2.0, 20.7)	4.2 (0.8, 20.2)	12.4 (4.3, 31.2)	29.2 (12.2, 55.0)	

n = column total, not adjusted for nonresponse using sample weights.

^a^ P-value from Rao-Scott chi-square test.

^b^ P-value from Rao-Scott chi-square test with assumed design correction of 2 (conservative estimate).

*Statistically significant at p = 0.05.

#### Immigration status

Immigration status at the time the questionnaire was completed was significantly associated with risk factors for HIV exposure and transmission, as shown in Table 4. Immigration status was significantly associated with having a history of forced or unwanted sex (χ_RS_^2^ = 27.54, df = 3, p < 0.001), ever testing for HIV (χ_RS_^2^ = 8.29, df = 3, p = 0.040) or ever mixing sex with drugs or alcohol (χ_RS_^2^ = 10.66, df = 3, p = 0.014). There were also trends with regards to the security of one’s immigration status. As security in immigration status increased (i.e. moving from “other” to “naturalized Canadian citizen”), the prevalence of: testing for HIV in the past 12 months increased, never having sex decreased, abstaining in the past 12 months decreased, having unprotected sex with a regular partner in the past 12 months increased, not using a condom in the past 12 months increased and having a history of STIs increased. Conversely, immigrants with the most unstable and insecure immigration statuses (i.e. those in the “other” category) reported a much higher prevalence of being in a non-monogamous sexual partnership in the past 12 months.

**Table 4:** Weighted prevalences for risk factors for HIV infection by immigration status

**Risk factors**	**Other^ (n = 21)**	**Permanent resident/landed immigrant or refugee (n = 38)**	**Naturalized Canadian citizen (n = 96)**	**Canadian-born (n = 29)**	**P-value**
	**wPrev (95% CI)**	**wPrev (95% CI)**	**wPrev (95% CI)**	**wPrev (95% CI)**	
*Factors associated with exposure to HIV*					
Age of sexual debut					0.516^b^
Never had sex	28.1 (13.2, 50.1)	19.9 (6.3, 47.6)	10.1 (5.4, 18.2)	6.9 (1.9, 21.9)	
12 years old or younger	21.6 (5.8, 55.2)	——	9.8 (3.3, 25.6)	5.3 (1.0, 23.4)	
13 to 15 years old	16.2 (4.9, 42.1)	5.3 (1.5, 17.3)	9.5 (3.8, 21.9)	17.1 (7.1, 36.0)	
16 to 18 years old	7.4 (1.8, 26.2)	27.4 (12.3, 50.3)	33.1 (22.3, 46.0)	31.3 (14.8, 54.4)	
19+ years old	20.5 (8.6, 41.6)	43.4 (19.8, 70.3)	22.5 (14.5, 33.1)	37.6 (18.2, 62.0)	
Engaged in transactional sex	10.1 (2.9, 29.6)	——	0.9 (0.14, 5.4)	8.5 (2.3, 26.6)	0.205^b^
History of forced/unwanted sex	16.7 (5.5, 40.9)	7.8 (2.7, 20.7)	13.7 (7.8, 23.0)	58.5 (37.2, 77.0)	**<0.001**^a^*
Had a sexual partner who injected drugs	——	——	0.7 (0.1, 5.0)	11.0 (3.7, 28.8)	0.105^b^
*Factors associated HIV exposure and transmission*					
Ever test for HIV	61.5 (38.9, 80.0)	80.2 (52.5, 93.7)	41.8 (29.8, 54.9)	72.0 (51.5, 86.1)	**0.040**^a^*
HIV test in Canada, past year	4.9 (0.9, 22.9)	16.2 (7.6, 31.3)	19.2 (10.7, 32.1)	7.4 (2.1, 22.6)	0.212^a^
Shared drug use equipment	——	——	——	4.2 (0.8, 19.5)	0.380^b^
Abstinence, lifetime	28.1 (13.2, 50.1)	19.9 (6.3, 47.6)	10.1 (5.4, 18.2)	6.9 (1.9, 21.9)	0.223^a^
Abstinence, past year	44.7 (24.2, 67.2)	35.6 (16.1, 61.3)	26.6 (17.2, 38.8)	10.0 (3.4, 25.9)	0.140^a^
Unprotected sex, cohabiting regular partner, past year	22.6 (9.1, 45.8)	50.9 (26.4, 74.9)	56.2 (42.9, 68.6)	44.9 (25.4, 66.2)	0.314^a^
Unprotected sex, non-cohabiting regular partner, past year	10.3 (3.0, 29.9)	44.9 (21.1, 71.2)	43.7 (31.4, 56.8)	38.1 (20.6, 59.3)	0.240^a^
Unprotected sex during last intercourse, regular partner	10.3 (3.0, 29.9)	47.0 (23.0, 72.4)	48.0 (35.3, 61.0)	25.8 (12.5, 45.9)	0.072^a^
Unprotected sex, casual partner, past year	7.5 (1.8, 26.3)	1.5 (0.2, 11.8)	14.8 (6.3, 31.0)	5.5 (1.3, 20.0)	0.090^a^
Unprotected sex during last intercourse, casual partner	——	27.7 (7.8, 63.5)	11.5 (4.3, 27.5)	5.5 (1.3, 20.0)	0.143^b^
Never using condom, past year	6.9 (1.5, 26.1)	27.4 (13.8, 47.1)	44.0 (31.3, 57.6)	43.6 (23.5, 66.0)	0.081^a^
Ever mixed sex with drugs or alcohol	33.8 (16.4, 57.2)	18.0 (7.9, 36.1)	31.7 (20.5, 45.6)	63.9 (40.4, 82.1)	**0.014**^a^*
Non-monogamous sexual partnership, past year	36.0 (15.3, 63.5)	7.6 (2.1, 23.7)	12.8 (5.4, 27.3)	16.6 (6.5, 36.0)	0.194^a^
History of sexually transmitted infections	13.3 (4.4, 33.4)	17.0 (7.0, 35.5)	20.8 (11.3, 35.0)	42.3 (23.1, 64.1)	0.358^a^
Number of sex partners, lifetime					0.135^b^
None	28.1 (13.2, 50.1)	19.9 (6.3, 47.6)	0.1 (5.4, 18.2)	6.9 (1.9, 21.9)	
1	——	24.0 (5.6, 62.5)	8.4 (4.3, 15.6)	——	
2 to 4	20.2 (8.4, 41.3)	20.4 (9.6, 38.0)	21.9 (13.8, 33.0)	6.6 (1.8, 21.5)	
5 to 9	18.3 (6.5, 41.8)	15.8 (5.6, 37.3)	20.3 (11.0, 34.3)	23.4 (9.2, 48.1)	
10 to 19	7.7 (1.9, 26.6)	4.9 (0.9, 22.6)	7.9 (3.4, 17.1)	34.3 (15.9, 59.1)	
20 or more	18.5 (4.1, 54.6)	2.0 (0.3, 12.5)	13.4 (5.9, 27.7)	14.4 (5.5, 32.6)	
Number of sex partners, past year					(p = 0.087)^a^
0	44.7 (24.2, 67.2)	35.6 (16.1, 61.3)	26.6 (17.2, 38.8)	10.0 (3.4, 25.9)	
1	15.5 (5.6, 36.0)	27.3 (13.2, 48.1)	40.0 (28.2, 53.0)	56.9 (34.6, 76.8)	
2	23.9 (7.2, 56.0)	26.0 (6.8, 62.7)	18.8 (10.7, 30.9)	3.9 (0.7, 17.8)	
3 or more	15.9 (5.1, 40.2)	6.1 (1.4, 22.7)	7.9 (2.5, 22.5)	29.2 (12.2, 55.0)	

n = column total, not adjusted for nonresponse using sample weights.

^a^ P-value from Rao-Scott chi-square test.

^b^ P-value from Rao-Scott chi-square test with assumed design correction of 2 (conservative estimate).

^Includes temporary workers, visitors, students and non-status individuals.

*Statistically significant at p = 0.05.

#### Employment status

Like immigration experience, employment status appears to have an important impact on HIV risk, as shown in Table 5. Employment status was significantly associated with: age of sexual debut (χ_RS_^2^ = 20.86, df = 8, p = 0.008), never having sex (χ_RS_^2^ = 13.03, df = 2, p = 0.002), abstaining in the past 12 months (χ_RS_^2^ = 6.28, df = 2, p = 0.043), having unprotected sex with cohabiting regular (χ_RS_^2^ = 6.14, df = 2, p = 0.047) and casual (χ_RS_^2^ = 9.92, df = 2, p = 0.007) partners, never using a condom in the past year (χ_RS_^2^ = 10.45, df = 2, p = 0.005), having a history of STIs (χ_RS_^2^ = 8.03, df = 2, p = 0.018), number of lifetime sex partners (χ_RS_^2^ = 27.46, df = 10, p = 0.002) and number of sex partners in the last year (χ_RS_^2^ = 26.44, df = 6, p < 0.001). Those with lower employment security (i.e. students and those who were unemployed or underemployed) appeared to be a lower risk for HIV exposure or transmission. Compared to those who held a regular full-time position or were self-employed, they were more likely to have abstained from sex in the past 12 months, less likely to have had unprotected sex in the past year, and less likely to have a history of STIs. However, they also had more sex partners in the past year than those who were self-employed or in regular, full-time employment.

**Table 5:** Weighted prevalences for risk factors for HIV infection by employment status

**Risk factors**	**Unemployed or underemployed^** **(n = 43)**	**Student** **(n = 78)**	**Employed in a regular full-Time position, or self-employed (n = 66)**	**P-value**
	**wPrev.(95% CI)**	**wPrev.(95% CI)**	**wPrev.(95% CI)**	
*Factors associated with exposure to HIV*				
Age of sexual debut				**0.008**^b^*
Never had sex	9.0 (3.5, 21.2)	29.1 (16.8, 45.6)	——	
12 years old or younger	4.5 (0.8, 21.1)	2.0 (0.5, 8.1)	14.4 (5.3, 33.8)	
13 to 15 years old	5.7 (1.8, 16.9)	8.9 (4.1, 18.5)	12.9 (5.1, 29.1)	
16 to 18 years old	22.1 (9.9, 42.2)	23.6 (13.2, 38.6)	38.9 (25.7, 54.0)	
19+ years old	47.2 (25.9, 69.5)	27.1 (15.6, 42.9)	24.3 (14.4, 38.0)	
Engaged in transactional sex	3.9 (1.0, 14.3)	1.4 (0.3, 7.7)	2.6 (0.5, 13.0)	0.719^a^
History of forced/unwanted sex	7.9 (2.8, 19.9)	31.3 (18.0, 48.6)	20.6 (11.8, 33.5)	0.079^a^
Had a sexual partner who injected drugs	——	2.4 (0.6, 8.7)	3.7 (1.0, 13.4)	0.620^b^
*Factors associated HIV exposure and transmission*				
Ever test for HIV	63.3 (42.6, 80.0)	59.4 (43.8, 73.3)	57.2 (41.3, 71.8)	0.403^a^
HIV test in Canada, past year	22.8 (8.8, 47.7)	18.6 (10.7, 30.2)	15.1 (6.5, 31.4)	0.636^a^
Shared drug use equipment	——	——	1.9 (0.3, 10.0)	0.557^b^
Abstinence, lifetime	9.0 (3.5, 21.2)	29.1 (16.8, 45.6)	——	**0.002**^b^*
Abstinence, past year	29.7 (15.1, 50.1)	36.8 (23.4, 52.5)	12.2 (6.3, 22.2)	**0.043**^a^*
Unprotected sex, cohabiting regular partner, past year	46.1 (25.3, 68.4)	35.3 (23.3, 49.3)	65.3 (48.7, 78.8)	**0.047**^a^*
Unprotected sex, non-cohabiting regular partner, past year	39.9 (19.9, 63.9)	26.7 (16.6, 39.9)	51.1 (35.8, 66.2)	0.109^a^
Unprotected sex during last intercourse, regular partner	43.7 (23.2, 66.7)	20.1 (12.3, 31.1)	53.9 (38.3, 68.7)	**0.014**^a^*
Unprotected sex, casual partner, past year	3.2 (0.7, 13.3)	4.2 (1.5, 11.2)	18.4 (7.5, 38.5)	**0.007**^a^*
Unprotected sex during last intercourse, casual partner	18.7 (4.6, 52.5)	5.6 (1.7, 16.7)	14.0 (4.8, 34.4)	0.343^a^
Never using condom, past year	26.6 (13.3, 46.2)	21.9 (11.8, 36.9)	55.2 (39.6, 69.9)	**0.005**^a^*
Ever mixed sex with drugs or alcohol	30.0 (14.0, 53.1)	29.2 (17.0, 45.3)	47.7 (32.7, 63.1)	0.305)^a^
Non-monogamous sexual partnership, past year	22.7 (8.5, 48.2)	15.1 (7.5, 28.0)	16.4 (6.8, 34.6)	0.734^a^
History of sexually transmitted infections	9.1 (3.3, 22.8)	14.1 (7.1, 26.1)	38.5 (24.4, 54.8)	**0.018**^a^*
Number of sex partners, lifetime				**0.002**^b*^
None	9.0 (3.5, 21.2)	29.1 (16.8, 45.6)	——	
1	23.7 (7.7, 53.7)	2.8 (0.8, 9.2)	1.9 (0.3, 10.0)	
2 to 4	17.5 (8.1, 33.7)	19.8 (11.9, 31.1)	18.2 (10.5, 29.7)	
5 to 9	17.3 (6.6, 38.1)	16.6 (7.8, 31.9)	21.5 (11.4, 36.9)	
10 to 19	16.8 (4.9, 44.0)	15.2 (6.0, 33.5)	13.8 (6.5, 26.9)	
20 or more	6.9 (2.0, 21.6)	3.4 (1.1, 10.1)	20.7 (9.9, 38.2)	
Number of sex partners, past year				**<0.001**^**a**^**c**
0	29.7 (15.1, 50.0)	36.8 (23.4, 52.5)	12.2 (6.3, 22.2)	
1	23.6 (11.8, 41.6)	27.9 (16.7, 42.8)	56.3 (40.4, 71.0)	
2	41.6 (20.6, 66.2)	11.7 (5.8, 22.0)	14.6 (6.5, 29.8)	
3 or more	2.1 (0.4, 11.8)	20.9 (10.1, 38.3)	9.9 (2.9, 28.6)	

n = column total, not adjusted for nonresponse using sample weights.

^a^ P-value from Rao-Scott chi-square test.

^b^ P-value from Rao-Scott chi-square test with assumed design correction of 2 (conservative estimate).

^ Includes those who do not fall in the other three categories, but are: unemployed, employed occasionally, employed seasonally, or employed part-time.

*Statistically significant at p = 0.05.

## Discussion

Perceptions about risk and actual behavioural risk in the ACB population converge in some areas, and diverge in others. First, while participants saw HIV risk as removed from Canada, quantitative data showed that behavioural HIV risk was higher among immigrants who had been in Canada longer and was higher among born Canadians than among immigrants. The comparatively low overall HIV prevalence in Canada may be responsible for this perception. Second, as per community members’ and service providers’ perceptions, sexual partner concurrency was fairly common in the ACB population. While there were no significant differences according to the chi-square tests comparing sexual partner concurrency in the different groups, it seems that women and people living at or below the LICO may be more likely to report being in a non-monogamous partnership. Third, abstinence was mentioned as a reason for low perception of risk by women, but Phase II showed that women were not significantly more likely than men to either have never had sex or be abstinent in the past year. In fact, never having sex was associated with poverty status, length of time in Canada and employment status. Furthermore, past year abstinence was significantly more likely to occur among: people living below the LICO, immigrants who had been in Canada for less time and people with less stable employment statuses. Fourth, Phase I participants said mixing sex with drugs or alcohol was a risk factor for HIV, and Phase II results show that women, immigrants who had been in Canada for less time, people with more stable immigration statuses, and people with less stable employment statuses were less likely to engage in this behaviour than other groups. Fifth, past year HIV testing was relatively low, and it was not associated with any of the markers of SSP in this paper. However, lifetime HIV testing might be higher than service providers perceived. Sixth, the prevalences of unprotected sex with regular and casual partners were high, thus confirming perceptions about unprotected sex being an issue within the ACB population. People living above the LICO and those who had regular full-time employment or were self-employed were more likely to engage in unprotected sex. Although not statistically significant, the results suggest that people who had been in Canada for more than 5 years and Canadian-born persons are more likely to engage in unprotected sex than new immigrants.

The data from Phase I show that there may be a disconnect between community members’ and service providers’ perceptions about HIV risk in the ACB population. Both groups agreed that unprotected sex, partner concurrency and low prevalence of HIV testing were important risk factors within the community. However, while community members spoke about abstinence as a protective factor, service providers did not seem to be aware that past-year and long-term abstinence were fairly common. Also, some of the barriers to protection that service providers cited (e.g. the need to be loved, cultural norms around disclosing information) were not mentioned by community members at all. These discrepancies may reflect service providers adopting a more analytical lens based on their overall observations versus community members sharing their individual experiences. Alternatively, these discrepancies may be due to service providers relying on research from the United States of America and other countries to inform their work. Consequently, they may not have contributed their own experiences and observations, but rather they could be repeating information from other service providers or researchers, or worse, they could be relying on stereotypes to inform their perceptions. Stereotypes and erroneous perceptions can be damaging to HIV prevention and care efforts, as they influence the types of actions that are taken to address HIV [8].

The data illustrate that poverty status, immigration experience and employment status are linked to the distribution of HIV risk and protective behaviours. The link between gender and HIV risk behaviours may be less apparent, because the effect of gender on HIV risk is likely dependent on its interaction with other markers of SSP, as Intersectionality Theory demonstrates [12]. According to the data, those with higher SSP may be at greater risk of HIV exposure or transmission when compared to those with lower SSP. This is not surprising because, the combination of multiple marginalizations can create unique SSPs that simultaneously limit and enhance one’s agency [13,47]. Hence, the combination of ACB identity and low SSP may protect an individual from engaging in particular HIV risk behaviours. Studies have also shown that early in an HIV epidemic, people with higher SSP are at greater risk for infection. However, as the epidemic matures and effective prevention interventions are designed, people with higher SSP are able to access and take advantage of the interventions. Hence people with lower SSP will begin to be at greater risk for infection, comparatively [48]. Additionally, the “healthy immigrant effect” may be at play, which could explain why newer immigrants have lower risk than those who have been in Canada longer and Canadian-born persons [49]. Furthermore, the data on immigration may reflect the effect of immigrants being exposed to HIV prevention messages in their home countries prior to immigration.

Given these findings, HIV prevention interventions should not be based on the assumption that low SSP automatically means high behavioural risk. Illustratively, fairly recent studies from Sub-Saharan Africa have shown that higher income [16,19], higher educational attainment [48], being employed [20] and being male [24,50] were associated with increased HIV risk, so these findings are not unusual. Paradoxically, British and North American studies show that HIV risk is associated with low income or poverty [51,52], low educational attainment [53], female sex [2] and immigration experience [27]. These contradictions are not surprising, however, as the impacts of SSP are context-specific and are influenced by governance, policies, cultures, and values [9]. At minimum, prevention interventions for ACB people locally, and possibly in other parts of Canada, should include consideration of gender, poverty status, immigration experience and employment status.

### Limitations

Since the qualitative analyses were descriptive and more in-depth exploration is beyond the scope of this paper, deeper meanings of, and connections between perceptions were not explored in more detail. Furthermore, social desirability bias may be present when data about sensitive topics, such as HIV risk, are self-reported. This type of bias occurs when participants give inaccurate responses that others will view favourably, and it is more likely to occur when data are collected in less-anonymous ways, such as through in-person interviews [54]. It could have accounted for some of the discrepancies between service providers’ and community member’s perceptions about HIV risk and protective behaviours within the local ACB population. The interview results should be interpreted cautiously.

As convenience sampling was used, Phase II of the study was subject to selection bias. However, non-response weights were applied to adjust for some of this bias. The sample’s size was smaller than the 384 participants required for a desired precision of 5%. Consequently, the confidence intervals for the prevalence estimates are wide, so the prevalence estimates should be interpreted in light of these wide ranges of plausible values. However, the study was adequately powered to detect statistically significant relationships, even with the wide confidence intervals. In all, 31% of the *χ*^2^ tests (31/100) were significant at the p = 0.05 level. The percentage of significant *χ*^2^ tests ranged from 15% for gender and immigration status to 50% for employment status. These percentages show that chance alone does not account for the results. Furthermore, the results demonstrate significant differences between groups and patterns in the distribution of risk behaviours by SSP.

Lastly, even though some aspects of Dillman’s “Tailored Design Method” were applied [40], only 32% of questionnaires were returned, which may be another source for selection bias. Other steps could have been taken to increase recruitment, such as: providing monetary incentives, further shortening the questionnaire, and having a web-based version of the questionnaire. Participants were given the option to request an interviewer to administer the questionnaire, but none requested one. Notably, the proportion of questionnaires returned is comparable to the proportion of participants who were successfully recruited into a similar study with East Africans in Toronto, Canada that offered monetary incentives and used interviewers [5].

## Conclusion

These results show that ACB people’s and service providers’ perceptions about HIV risk differ and may be inconsistent with actual risk among ACB people. Furthermore, HIV risk behaviours are distributed according to markers of SSP, which make these SDOH important factors in the design of effective prevention interventions. Due to these risk perceptions, many HIV prevention interventions for ACB people in Ontario focus on women, low-income people, new immigrants and students. This study’s results suggest that this focus may be misplaced, and prevention interventions should at least target a broader cross-section of ACB people. On the other hand, these data might reflect the effectiveness of current interventions targeting women, students and new immigrants.

## Abbreviations

ACB: African, Caribbean and other Black; AIDS: Acquired Immune Deficiency Syndrome; BLACCH Study: Black, African and Caribbean Canadian Health Study; CBR: Community-based research; HIV: Human immunodeficiency virus; LICO: Low-income cut-off; QCA: Qualitative content analysis; SDOH: Social determinants of health; SSP: Social status and position; STI: Sexually transmitted infection.

## Competing interests

The authors declare that they have no competing interests.

## Authors’ contributions

SB designed the study, participated in data collection, designed and conducted the analyses and wrote the manuscript. GB supervised SB’s work on this study, provided feedback on the concept sheet for this study, and provided comments and ideas that were incorporated into the final manuscript. KNS and EL provided feedback that helped develop the study, guidance on research methods used, and comments and ideas that were used to develop the final manuscript. The BLACCH Study Team participated in designing the study, data collection and data entry. All authors read and approved the final manuscript.

## Pre-publication history

The pre-publication history for this paper can be accessed here:

http://www.biomedcentral.com/1471-2458/13/184/prepub
